# Small-scale evaluation of the efficacy and residual activity of alpha-cypermethrin WG (250 g AI/kg) for indoor spraying in comparison with alpha-cypermethrin WP (50 g AI/kg) in India

**DOI:** 10.1186/s12936-015-0739-7

**Published:** 2015-05-29

**Authors:** Sreehari Uragayala, Raghavendra Kamaraju, Satyanarayana Tiwari, Sushanta Kumar Ghosh, Neena Valecha

**Affiliations:** National Institute of Malaria Research Field Unit, Nirmal Bhawan-ICMR Complex, Poojanahalli, Kannamangala (Post), Devanahalli Taluk, Bengaluru, India; National Institute of Malaria Research, Sector 8, Dwarka, New Delhi India

**Keywords:** Alpha-cypermethrin, *Anopheles stephensi*, Indoor residual spraying, Residual efficacy

## Abstract

**Background:**

Indoor residual spraying (IRS) with different formulations of insecticides is being used for the control of mosquito vectors in many countries. In the present study, residual efficacy and duration of effectiveness of IRS with alpha-cypermethrin WG-SB (250 g AI/m^2^) formulation was compared with WP formulation (50 g AI/kg) in a small scale (Phase II) field trial.

**Methods:**

Two dosages, i.e. 20 and 30 mg AI/m^2^, were used and the efficacy and duration of effectiveness was assessed on alpha-cypermethrin susceptible population of *Anopheles stephensi*. Four types of surfaces were selected, namely cement wall with distemper coating, cement wall with lime coating, mud wall with lime coating, and brick wall unpainted. Spraying was carried out with Hudson sprayer fitted with control flow valve. Bioassays were carried out at weekly and then fortnightly intervals. Chemical analysis of filter paper samples collected from the sprayed surfaces was done at Walloon Agricultural Research Institute, Gembloux, Belgium.

**Results:**

Alpha-cypermethrin WG-SB showed residual efficacy (>80 % mortality) for 13–15 weeks for the 20 mg/m^2^ dosage and 13–16 weeks for the 30 mg/m^2^ dosage, whereas alpha-cypermethrin WP showed residual efficacy for 11–15 weeks for the 20 mg/m^2^ dosage and 11–14 weeks for the 30 mg/m^2^ dosage on the surfaces tested. The average of the applied to target dose ratio ranged from 0.89 to 1.17 for alpha-cypermethrin WG-SB at 20 mg AI/m^2^, 0.83–1.80 for the WG-SB at 30 mg AI/m^2^, 0.87–1.66 for alpha-cypermethrin WP at 20 mg AI/m^2^, and 0.68–1.06 for WP at 30 mg AI/m^2^. No adverse events were reported, either by the spray men or the household inhabitants, during and after the spray operations.

**Conclusions:**

The results suggest that the dose of WG 30 mg/m^2^ gave slightly longer effective residual action against malaria vector (16 weeks) on most common indoor surfaces and could be used for effective control of *Anopheles* mosquitoes. The WG formulation was found to be easy to handle, no smell was reported during the spraying and was found to be operationally acceptable for indoor residual spraying.

## Background

Indoor residual spraying (IRS) with insecticides is the main vector control strategy in the National Vector-borne Disease Control Programme (NVBDCP) in India to control malaria transmission [1]. For IRS, pyrethroids are used in many parts of the country endemic for malaria. The pyrethroids are usually available in wettable powder (WP) formulations. Pesticide manufacturers are innovating to produce formulations that are safe in storage, handling and spraying operations in field. The pesticide formulation is important in improving the properties of a chemical for handling, storage, application and may substantially influence effectiveness and safety [2]. Nowadays, there is a great need for new formulations which are cleaner and safer for the user, have minimal impact on the environment, and can be applied at the lowest dose rate. Controlled release formulations, like microencapsulation, wettable granules, capsule suspensions, are being developed to minimize the exposure during spray preparations, handling, slow release to extend the bioavailability of the insecticide on the surface, extended efficacy and to minimize the environment contamination, operational feasibility, storage, etc. Further, controlled release formulations have the ability to improve pesticide selectivity [3]. It is realized that water-dispersible granule (WG) formulation of pyrethroids offers such an alternative.

Alpha-cypermethrin is one among the insecticides recommended by World Health Organization (WHO) for IRS [4]. Its WP and SC (suspension concentrate) formulations have been tested by WHO in field conditions [5]. In trials carried out in Pondicherry (now Puducherry), India, alpha-cypermethrin WP sprayed indoors at dosage of 100 mg AI/m^2^, significantly reduced density of *Culex quinquefasciatus* and *Anopheles subpictus* with residual efficacy of 18–27 weeks on different surfaces such as cement, mud and thatch [6]. Lien et al. [7] reported efficacy of alpha-cypermethrin WP sprayed at 20 mg AI/m^2^ against *Aedes aegypti* in Taiwan. In Burkina Faso, alpha-cypermethrin was effective at 0.1 g AI/m^2^ dose against mosquito vectors [8]. Village-scale trials in The Philippines with alpha-cypermethrin WP IRS at 30 mg AI/m^2^ showed reduction in vector densities, parity rate and malaria incidence with an observed bio-efficacy on different sprayed surfaces (>90 %) for seven months [5]. A community randomized IRS trial in Pakistan, with alpha-cypermethrin WP and SC formulations at 25 mg AI/m^2^ showed reduced mosquito densities, and malaria prevalence with residual efficacy of four months against *Anopheles culicifacies* and *Anopheles stephensi* [9]. Alpha-cypermethrin WP is in use in India for vector control in rural and peri-urban areas. In villages in Karnataka State, India, synthetic pyrethroids are in use for vector control, which includes deltamethrin and alpha-cypermethrin.

In the present study, residual efficacy of alpha-cypermethrin WG-SB (250 g AI/kg) IRS in comparison with alpha-cypermethrin WP (50 g AI/kg) was assessed on most common wall surfaces, namely cement, mud and brick using a susceptible strain of *An. stephensi* in bioassays. The main objective of this study was to determine the persistence of alpha-cypermethrin WG-SB formulation on local indoor surfaces against *An. stephensi* in comparison to WP formulation.

## Methods

### Insecticides

Alpha-cypermethrin WG-SB, a water-dispersible granular formulation packed in water-soluble bags (WG-SB, brand name RUBI 250 WG) containing 250 g AI/kg active ingredient (Batch No. AG-01/13, date of manufacture May 2013) and sachets of alpha-cypermethrin WP formulation containing 50 g AI /kg active ingredient (Batch No. Lot-01, manufacture date February 2013) were supplied by M/s Tagros Chemicals India Pvt. Ltd, Chennai, India. Two dosages of each formulation were used in the study, 20 and 30 mg AI/m^2^.

### Study area

The study was conducted in Balepura village (13.19° N, 77.77° E) of Nalluru PHC, Devanahalli Taluka, in Karnataka state, India. The proposed study village is located in an irrigated rural area. The village has not received IRS in the last five years. There are around 250 houses in the village. The climate of the area is tropical and broadly the seasons are monsoon (July to October), winter (November to February) and summer (March to June). The temperature ranges from 15 to 23 °C in winter and 25 to 35 °C in summer.

Houses in the villages are of various types: mud/brick, cement brick walls with mostly mud-plastering, cement-plastering with lime-coating or cement-plastering with distemper coating. Cattle-sheds are generally either separate enclosures adjacent to the houses or are mixed dwellings, i.e., share a common roof with human dwelling rooms. The inhabitant human population is stable and the major occupation of the people is agriculture. Major crops grown in the area are rice, millet, and vegetables.

### Baseline susceptibility of *Anopheles stephensi* to alpha-cypermethrin

For susceptibility tests, insecticide susceptible strain of *An. stephensi* mosquitoes maintained in the insectary at National Institute of Malaria Research (NIMR) Field Unit were used, quarterly tested for insecticide susceptibility against DDT, malathion and pyrethroids using WHO test kit [10]. Anopheles larvae were collected from different localities prior to the study and were colonized. F_1_ generation of the mosquitoes were tested for the insecticide susceptibility. *Anopheles stephensi* adults susceptible for alpha-cypermethrin were used for all cone bioassay tests.

### Selection of houses and rooms in the study villages and informed consent

The study obtained consent of the *Panchayat Sarpanch* (head of the village council) and local opinion leaders. Consent of the PHC Medical Officer, Nalluru was obtained to undertake the spraying operations in the village and requested not to undertake any spraying operation in the village during the study period. Altogether, 69 houses were selected for the present study after obtaining consent from the household heads. The houses were allotted as per the protocol and a minimum of three houses were sprayed with each dose and formulation.

Houses were selected based on the category and type of walls. Altogether, 61 houses were selected for spraying in the present study along with eight control surfaces. Four types of wall surfaces were selected, namely cement wall with distemper coating (a paint containing water, chalk powder and glue/resin), cement wall with lime coating, mud wall with lime coating and brick wall unpainted.

Written informed consent of the head person of the potential households was obtained using *Kannada* (local dialect) version of the informed consent form developed by NIMR.

### Spraying of insecticides indoors and information sheet for spray men

The spray men were given two-day training in spraying technique prior to spraying operation in the study village. These spray men were employed under supervision to spray the assigned households with the given formulation and dosage. Informed consent was obtained from the spray men and they were briefed about the objectives of the project and necessary instructions were given about the safe handling, spraying and precautions to be taken to avoid accidental exposure to insecticide. Protective clothing, goggles, gloves, etc., were provided to the spray men for their general safety. They were briefed on possible adverse effects and the need to fully comply with safety instructions to avoid adverse effects.

Only one round of spraying was done using hand-operated compression sprayer (Hudson X-Pert Sprayer, procured from M/s. H.D. Hudson Asia Limited, Hong Kong) fitted with pressure gauze, a control flow valve (set at 1.5 bar) and a flat fan nozzle (8002) according to the standard WHO application procedure [11]. Spraying was done at 58 psi working pressure in the sprayer tank. Calibration of the spray pumps was done to obtain uniform and good quality spraying for the targeted dose. Participating households were informed well in advance about the spraying schedule. The spraying operation was undertaken from 20 November, 2013. Each dose was sprayed on single day and the spraying was completed in four days. The same person sprayed all the selected houses to avoid bias. Medical Officer of the Nalluru PHC accompanied the team during the days of spraying to attend to complaints, if any. The Medical Officer conducted physical examination and interviewed the inhabitants as well as spray men every day after spraying.

### Assessment of the quality of treatment

To assess the quality and accuracy of insecticide treatment on indoor surfaces in each house, three rectangular pieces of Whatman® filter paper No. 1 (size 10 cm x 10 cm) were attached on different surfaces at various heights using fine steel pins. Three filter papers in each sprayed house were affixed. These were placed at three different heights of the walls. One day after spraying, all the filter paper samples were collected and packed in aluminium foil and labelled according to the dose, formulation and type of surface and stored in a refrigerator at 4 °C. Six to nine samples for each dose and type of surface were selected randomly for chemical analysis. Later, these were sent to Walloon Agricultural Research Institute, Belgium for chemical analysis of the insecticide residue (*n* = 97). The spots where filter papers were affixed were marked with pencil to avoid exact placement of cones on them during subsequent cone bioassay tests.

### Contact bioassays

Bioassays were conducted one week after spraying and at regular intervals in the houses sprayed. For bioassay, the cones were attached on the surfaces using adhesive tape and batches of ten mosquitoes were released gently. After 30 min of exposure to the sprayed surface, mosquitoes were removed gently from the cones and kept in plastic cups covered with a netting piece. Mosquitoes were provided with cotton wool moistened with 10 % glucose solution and kept at 28 ± 2 °C temperature and >80 % RH (relative humidity). Knock-down was recorded after 60 min and mortality was scored 24 h post-exposure. Data were recorded in a structured proforma for further analysis. Subsequent bioassays were done on a close spot but not exactly on the same spot. Care was taken to include all houses for bioassays in each visit. Bioassays were terminated when the mortality was below 80 % in two consecutive bioassays. Mortalities in test replicates were corrected by applying Abbott’s formula [12] when the mortalities in control replicates were between 5 and 20 %.

### Human and environmental safety

At the time of taking informed consent from the participating households, they were educated to take safety precautions to avoid any possible risk during and after the spraying of their houses. On the day of spraying all family members were again advised by a spray team supervisor to remain out of the rooms during the spray and up to two to three hours after spraying. The adult householders present at the time of spraying were advised to tell their children not to intentionally touch the sprayed walls for at least one day after spraying since the walls remained wet for about a day. Further, they were informed not to paint, white wash, or plaster the sprayed surfaces till the completion of the study.

For environmental safety, used pesticide containers or sachets were disposed off using correct procedure [13]. As the product was supplied in a plastic sachet, the used sachets were buried in a deep pit.

### Adverse effects on spray men

An assessment of the adverse effects was made using a questionnaire. All the spray men and supporting staff participated in spraying operations were interviewed at the end of the day of spraying by the Medical Officer of the Nalluru PHC, and again in the following morning and one week later. Adverse events reported were recorded. Assessment of adverse events on inhabitants of the sprayed houses/rooms was also done after one and four weeks of the spraying using a questionnaire by the Medical Officer as well as investigator and NIMR staff.

### Data analysis

Data were entered in MS Excel. Statistical analysis was done using generalized linear mixed model with binomial link function probability distribution. Comparisons were made between dosages, surfaces and duration of effectiveness. Statistical analysis was done using SPSS 20.0 (SPSS Inc. Chicago). Data were adjusted to nearest weeks in few cases to fit into the model. Mortality data up to sixteen weeks were included in the generalized linear mixed model for uniformity and comparison.

### Ethical and institutional clearances

Ethical clearance of the study was obtained from the Ethics Committee of NIMR, New Delhi. The Research Advisory Committee, Scientific Advisory Committee of NIMR approved the protocol. Health Minister’s Screening Committee of the Ministry of Health & Family Welfare, Government of India approved the project to be undertaken in India.

## Results

**Fig 1:**
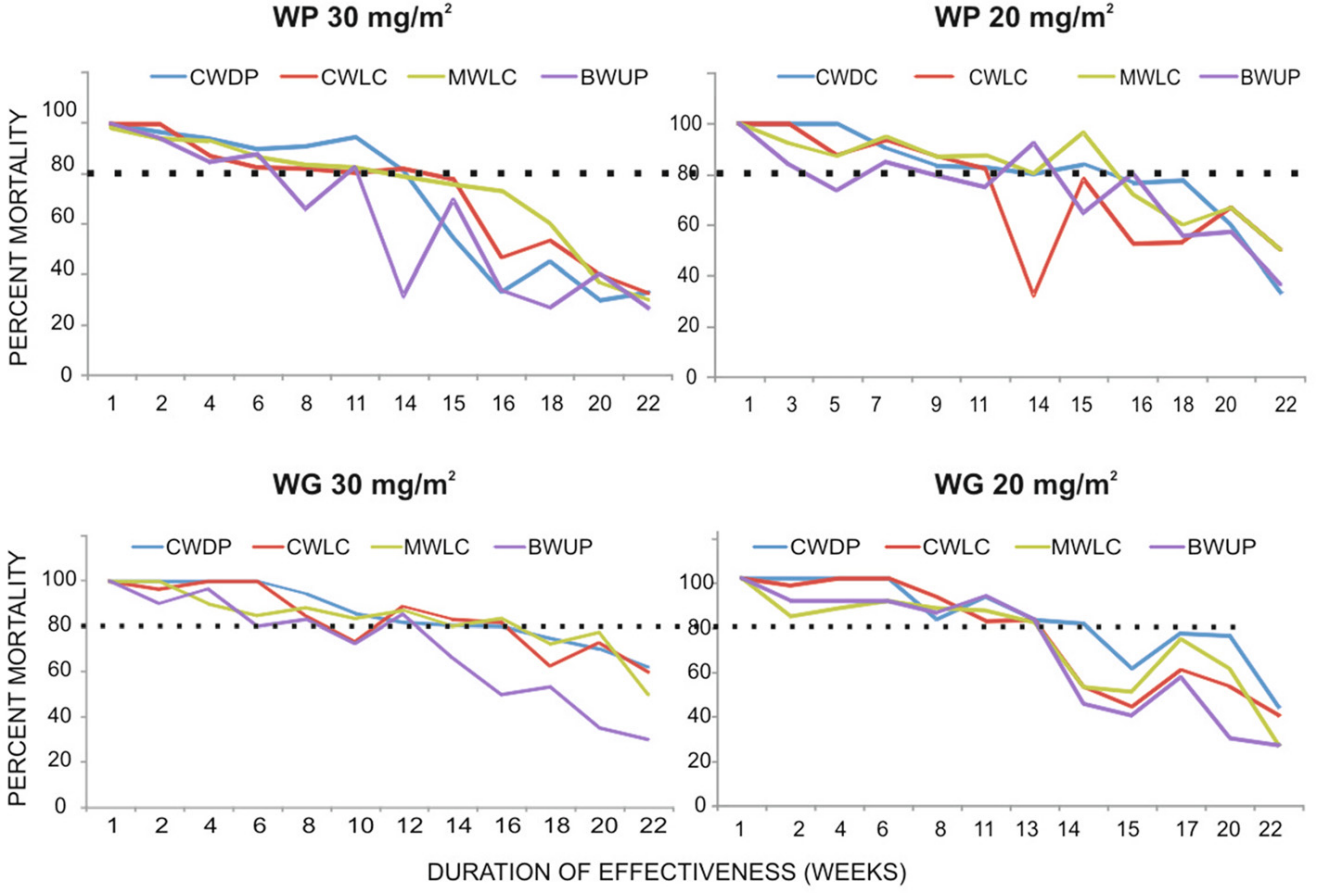
The duration of effective residual action (>80 % mortality in cone bioassays) of different dosages and formulations of alpha-cypermethrin against *Anopheles stephensi* (dotted line represents 80 % cut off; CWDP- Cement wall distemper coated, CWLC- Cement wall lime coated, MWLC- Mud wall lime coated, BWUP- Brick wall unpainted)

Results of cone bioassays for assessing effectiveness and duration of effectiveness on WG 30 mg/m^2^ dose sprayed surfaces are shown in Fig. 1. The results revealed that more than 80 % mortality was reported up to 16 weeks on cement wall + distemper coated, cement wall + lime coated, and mud wall + lime coated surfaces. In contrast, the residual efficacy was up to 13 weeks on brick wall + unpainted surface.

The results of cone bioassays on alpha-cypermethrin WG 20 mg/m^2^ dose sprayed surfaces are shown in Fig. 1. The results show that the duration of effectiveness of alpha-cypermethrin in producing more than 80 % mortality in *An. stephensi* was 15 weeks on cement wall + distemper coated surface, 13 weeks on cement wall + lime coated, mud wall + lime coated and brick wall unpainted surfaces.

Results of cone bioassays on different surfaces sprayed with WP formulation 30 mg/m^2^ dose are shown in Fig. 1. The results revealed that the duration of effectiveness (>80 % mortality in test mosquitoes) of alpha-cypermethrin 30 mg/m^2^ was 14 weeks on cement wall with distemper coating and cement wall + lime coated surfaces, 11 weeks on mud wall with lime coating and brick wall unpainted surfaces. There was a significant differences in mortalities on different surfaces. Results of bioassays on different surfaces sprayed with WP formulation 20 mg/m^2^ dose are shown in Fig. 1. The results revealed that the duration of effectiveness (>80 % mortality in test mosquitoes) of alpha-cypermethrin 20 mg/m^2^ was 15 weeks post-spraying on cement wall + distemper coated surface and mud wall + lime coated surface, 11 weeks on cement wall + lime coated and brick wall unpainted surfaces. .

There is no clear evidence to show that the residual efficacy of alpha-cypermethrin 250 WG differed from alpha-cypermethrin 50 WP when applied at 20 and 30 mg/m^2^. The average of the applied to target dose ratio was 0.89-1.17 for alpha-cypermethrin WG at 20 mg AI/m^2^, 0.83-1.80 for the WG at 30 mg AI/m^2^, 0.87-1.66 for alpha-cypermethrin WP at 20 mg AI/m^2^, and 0.68-1.06 for WP at 30 mg AI/m^2^. The alpha-cypermethrin content variation between filter papers, as expressed as relative standard deviation (RSD), ranged from 16.1 to 85.3 % (Table 1). The average applied to target dose ratio was within the expected range of the target dose ±25 % for the WG-SB treatment with the exception of 30 mg/m^2^ treatment on one surface (mud wall with lime coating), which was higher than expectation (Table 1). The average applied/target dose ratio ±25 % for the WP treatment was higher than expected for three of the four surfaces at 20 mg/m^2^ and below expectation for one of the surfaces at 30 mg/m^2^.

**Table 1:** Results of chemical analysis of filter samples collected from sprayed houses

Formulation	Surface type	Target dose (mg/m^2^)	Applied dose (mg ai/m^2^)	No. filter-paper samples analysed	Applied/target ratio	D	RSD
WP	Cement wall + distemper coated	20	30.5	6	1.53	5.87	19.3
	Cement wall + lime coated	20	17.3	6	0.87	5.66	35.6
	Mud wall + lime coated	20	29.8	6	1.5	4.8	16.1
	Brick wall unpainted	20	33.06	6	1.66	7.4	22.4
	Cement wall + distemper coated	30	20.55	6	0.68	7.5	36.7
	Cement wall + lime coated	30	26.55	6	0.88	11.6	43.8
	Mud wall + lime coated	30	22.8	6	0.76	12.2	53.6
	Brick wall unpainted	30	31.7	6	1.06	13.5	42.5
WG	Cement wall + distemper coated	20	17.7	6	0.89	8.4	47.5
	Cement wall + lime coated	20	23.3	6	1.17	5.76	24.7
	Mud wall + lime coated	20	22.8	6	0.9	15.3	85.3
	Brick wall unpainted	20	21.7	7	1.11	3.7	16.9
	Cement wall + distemper coated	30	26.45	6	0.83	11.1	44
	Cement wall + lime coated	30	30.8	6	1.03	11.9	38.7
	Mud wall + lime coated	30	53.85	6	1.8	10.9	20.3
	Brick wall unpainted	30	26.4	6	0.88	15.1	57.4

**Fig 2:**
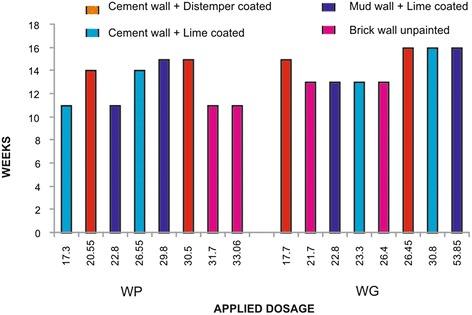
Applied dose as assessed from chemical analysis of filter papers *versus* > 80 % mortality reported on different sprayed surfaces (duration of effectiveness in weeks) with different formulations

A decrease in residual activity with time was observed. Some variations could be observed with WP formulation in spraying the target dose, which was also clearly reflected in bioassays. The bioassay results also coincided with the applied to target ratio (Fig. 2). Statistical analysis revealed that there is a significant difference in mortalities reported on different types of surfaces in all treatments and brick wall unpainted surface showed least efficacy in comparison to other types of surfaces (p < 0.000). In few cases there as a significant difference between mud wall lime coated and cement wall lime coated surfaces (p < 0.05). In most of the treatments, cement wall distemper coated surface showed higher efficacy than the other types. There was no significant difference when the WP20 mg and WG 20 mg doses were compared in between (p > 0.05). This indicates that both formulations were equally effective. Further, no significant difference was observed when the WP 20 mg and 30 mg doses were compared (p > 0.05) for all the surfaces tested. This may be probably due to variations in applied to target ratios.

### Human safety evaluation

Altogether, 48 household heads were interviewed after one week of spraying. None of the households reported any adverse events such as headache, itching, nausea, vomiting, suffocation, etc. The Medical Officer of Nalluru PHC supervised the spraying operations and conducted physical examination of the spray men one day after each spraying schedule. One supporting staff complained of headache for one hour and it subsided without any medication. Sneezing was reported in one spray man while spraying WP formulation for few minutes and it subsided after a while. Besides this, no adverse events were reported. Many inhabitants enthusiastically asserted the effectiveness of the spraying and reported that many insects such as cockroaches, ants, spiders, etc. were lying dead post-spraying and mosquito nuisance has been reduced significantly in their houses.

## Discussion

In the present study, the duration of effectiveness of IRS with alpha-cypermethrin WP and WG formulations was tested on most common indoor surfaces against *An. stephensi*. A dose of 30 mg AI/m^2^ could be effective up to 16 weeks on most common surfaces such as mud wall and cement wall. The results of this study conform to other studies that reported similar efficacy of alpha-cypermethrin IRS. In a study by Ratovonjato et al. [14] in Madagascar, comparing the efficacy of IRS with DDT, alpha-cypermethrin and deltamethrin in different settings, reported that IRS with pyrethroids did produce superior efficacy in terms of many entomological and parasitological indicators than IRS with 75 % DDT. In another study by Faraj et al. [15], using IRS with alpha-cypermethrin SC formulation sprayed at a dosage of 30 mg AI/m^2^ reported significant reduction in sandfly density as well as cutaneous leishmaniasis incidence after the spraying in northern Morocco, an endemic area for leishmaniasis.

In a study conducted in India with alpha-cypermethrin WP IRS at a dosage of 100 mg AI/m^2^, the efficacy lasted for 20 weeks against *Cx. quinquefasciatus* and *An. stephensi* in bioassays conducted on mud, cement and thatched surfaces [6]. A study conducted in China with alpha-cypermethrin WP impregnated bed nets, showed a good mass killing effect on the vector population [16]. In a study carried out in The Gambia using alpha-cypermethrin SC in comparison to permethrin EC [emulsifiable concentration] and lambda-cyhalothrin EC formulations, unwashed nets impregnated with alpha-cypermethrin were significantly more effective at killing anopheline mosquitoes in bioassays than nets impregnated with permethrin or lambda-cyhalothrin [17]. In a WHOPES [World Health Organization Pesticide Evaluation Scheme] supervised Phase II IRS trial with alpha-cypermethrin WP formulation at the rate of 100 mg/m^2^ dose, bioassay results showed 76 % immediate mortality and 24 % and delayed mortality in *Anopheles* mosquitoes with remarkable knock-down effect [5].

In a WHOPES supervised trial in The Philippines with alpha-cypermethrin WP IRS, contact bioassays, using WHO standard cones and wild-caught *Anopheles flavirostris* blood-fed females gave 100 % mortality up to seven months, on all treated surfaces, except cement (90 %) [5]. In a Phase III trial in Pakistan, alpha-cypermethrin WP and SC formulations sprayed at a dosage of 25 mg AI/m^2^ significantly reduced density of *Anopheles* mosquitoes and *vis a vis* malaria transmission, indicating effectiveness of alpha-cypermethrin IRS [9]. A study of IRS with alpha-cypermethrin at a dosage of 50 mg/m^2^ in the Democratic Republic of São Tomé and Príncipe reported rapid reduction in malaria cases in children < nine years of age before and after the first IRS cycle; and also wood surfaces showed higher bio-efficacy than cement surface [17]. All these trials reported the effectiveness of alpha-cypermethrin IRS against *Anopheles* mosquitoes and in the present study alpha-cypermethrin WG formulation showed efficacy up to four months against *An. stephensi* in bioassays. It is interesting to note that the corrected mortality in most of the surfaces sprayed with WG formulation (both 30 and 20 mg/m^2^ dosages) was >60 % up to 22 weeks of evaluation. The WG formulation was found to be more efficacious and operationally feasible than the WP formulation. The observed variations in the duration of effectiveness is due to differences in applied to target dose as revealed from chemical analysis. Further, WG-SB formulation was easy to handle and there are limited chance of direct exposure, mixing of contents was easy and miscibility was higher than the WP formulation. The most important part was achieving the target doses, which was achieved using a controlled flow valve.

Alpha-cypermethrin, a synthetic pyrethroid possessing highly irritating properties [18,19] and immediate toxicity effect, is a suitable insecticide for IRS. Alpha-cypermethrin, is primarily a contact irritant and a toxicant. In a study it was demonstrated that alpha-cypermethrin exhibited exiting response and high knock-down in laboratory assays and inside huts. However, this compound did not elicit a repellent response from the mosquitoes under controlled laboratory conditions or repel mosquitoes from entering huts in the field [19].

## Conclusion

The results suggest that in areas where malaria transmission is seasonal, especially in monsoon and post-monsoon months lasting for four to six months, one round of IRS with alpha-cypermethrin WG 30 mg/m^2^ dose shall be effective in controlling malaria transmission, provided good applied to target dose ratio is achieved. However, more studies are indicated to study the epidemiological impact of one round of IRS with alpha-cypermethrin IRS in controlling malaria in areas with seasonal transmission.
